# Structural Adaptation and Physiological Mechanisms in the Leaves of *Anthyllis vulneraria* L. from Metallicolous and Non-Metallicolous Populations

**DOI:** 10.3390/plants9050662

**Published:** 2020-05-23

**Authors:** Marzena Sujkowska-Rybkowska, Ewa Muszyńska, Mateusz Labudda

**Affiliations:** 1 Department of Botany, Institute of Biology, Warsaw University of Life Sciences-SGGW, Nowoursynowska 159, Building 37, 02-776 Warsaw, Poland; ewa_muszynska@sggw.edu.pl; 2 Department of Biochemistry and Microbiology, Institute of Biology, Warsaw University of Life Sciences-SGGW, Nowoursynowska 159, Building 37, 02-776 Warsaw, Poland; mateusz_labudda@sggw.edu.pl

**Keywords:** antioxidants, apoplast, cell wall, calamine, kidney vetch, trace metals

## Abstract

Calamine wastes highly contaminated with trace metals (TMs) are spontaneously inhabited by a legume plant *Anthyllis vulneraria* L. This study determined an adaptation strategy of metallicolous (M) *A. vulneraria* and compared it with that of the non-metallicolous (NM) ecotype. We hypothesized that TMs may lead to (i) leaf apoplast modifications and (ii) changes in the antioxidant machinery efficiency that facilitate plant growth under severe contamination. To verify our hypothesis, we implemented immunolabelling, transmission electron microscopy and biochemical measurements. NM leaves were larger and thicker compared to the M ecotype. Microscopic analysis of M leaves showed a lack of dysfunctions in mesophyll cells exposed to TMs. However, changes in apoplast composition and thickening of the mesophyll and epidermal cell walls in these plants were observed. Thick walls were abundant in xyloglucan, pectins, arabinan, arabinogalactan protein and extensin. The tested ecotypes differed also in their physiological responses. The metallicolous ecotype featured greater accumulation of photosynthetic pigments, enhanced activity of superoxide dismutase and increased content of specific phenol groups in comparison with the NM one. Despite this, radical scavenging activity at the level of 20% was similar in M and NM ecotypes, which may implicate effective reduction of oxidative stress in M plants. In summary, our results confirmed hypotheses and suggest that TMs induced cell wall modifications of leaves, which may play a role in metal stress avoidance in *Anthyllis* species. However, when TMs reach the protoplast, activation of antioxidant machinery may significantly strengthen the status of plants naturally growing in TM-polluted environment.

## 1. Introduction 

Mining activities in southern Poland introduce large quantities of waste highly contaminated with trace metals (TMs), which when occurring in high amount in the soil can be extremely toxic to plants, microorganisms and humans [1]. Young waste heaps are devoid of vegetation not only due to high concentrations of TMs but also because of strong insolation, winds, physiological drought and low nutrient content [2]. Vegetation in such unfriendly areas is necessary to reduce the spread of pollution but its establishment is a long-term process achievable only for selected plant species [1,3,4,5,6]. Old calamine wastes contaminated with Zn, Pb and Cd in Bolesław near Olkusz (southern Poland) are particularly suitable to investigate plant adaptation to trace metal pollution [1,6,7]. Although the habitat conditions are extreme, legume plants spontaneously colonize these sites [1,7,8,9]. 

Plants growing in contaminated areas take up metals mainly through their root system. When TMs reach shoots, they may alter the plant structure, physiology and metabolism [10,11]. Primary plant response to TMs includes rapid production of reactive oxygen species (ROS) and ROS-induced changes in the cellular redox state [11,12,13,14,15,16]. These reactive species are produced mostly in subcellular locations such as the nucleus, mitochondria, chloroplast, peroxisome, plasma membrane and the cell wall (CW) [11,12,13,14,15,16,17]. ROS can oxidize lipids, proteins or nucleic acids, leading to cell wall remodelling, lipid peroxidation and destruction of the cell membrane and ultrastructure [11,12,13,14,15,16,17]. TM-induced excess of ROS restricts plants’ ability to efficient photosynthesis by causing deformations of chloroplast ultrastructure [17,18]. ROS level is controlled by the rate of their production and elimination by the antioxidant system [11,12,13,14,15,16,17]. The plant antioxidant system includes enzymes (e.g., superoxide dismutase—SOD, catalase—CAT and peroxidase—POX) and non-enzymatic antioxidants controlling the lifetime of ROS, which are crucial for maintaining the functionality of plants [14,18,19,20]. The non-enzymatic antioxidant systems include phenols such as flavonoids, phenylpropanoids, anthocyanins and carotenoids that are strong antioxidants and metal chelators [14,18,19,20].

The mechanisms of stress avoidance in metal tolerant plants consist of reducing metal penetration into the protoplast, as well as sequestering ions in the apoplast, vacuoles, epidermis or trichomes [21]. The apoplast (all the compartments beyond the plasmalemma, including the cell wall) participates in stress perception and defence [22,23]. The cell wall is the first barrier that comes in contact with toxic ions and prevents TM transfer into the protoplast [23]. Although the cell wall structure and modifications were thoroughly analysed in the roots of plants exposed to TMs [22,23,24], they remain practically unexplored in the leaves. The cell wall structure, comprising of cellulose microfibrils and non-cellulosic neutral polysaccharides embedded in pectin matrix with proteins and phenolic compounds, confers TMs’ binding ability [22,23,24]. Excess accumulation of metals in the wall often leads to its stiffening and thickening [22,23,24]. One of the most important cell components are pectins, which, in terms of structure, can be classified into five types: homogalacturonan (HG), xylogalacturonan, rhamnogalacturonan I, rhamnogalacturonan II and arabinogalactan [22]. Pectins are synthesized in the Golgi apparatus and secreted into the apoplast as highly methylesterified forms. De-esterified pectins of the cell wall are considered the main compounds responsible for TM binding and immobilization since they contain negatively charged carboxylic groups with high affinity to TMs [22,23]. Such chemical structure makes pectin-saturated cell walls more stiff, which alters cell elongation and growth [22,24]. Other cell wall compounds such as structural proteins (arabinogalactans and extensins) and phenolics can modulate cell wall extensibility and also protect the plant against TMs [23,24]. In short, the cell wall may sequester TMs, but this may affect its synthesis, composition and mechanical properties. For example, exposure to TMs may increase the accumulation of polysaccharides, glycoproteins and lignin in root cell walls [25,26]. Changes occurring in cell walls in the presence of TMs are crucial to the cell defence against toxic ion penetration. Strengthening of its mechanical barrier properties represents adaptive improvements reducing apoplastic metal ion transport [23,24,25,26]. 

Legumes spontaneously occurring on TM-contaminated sites are of great interest to researchers due to their symbiotic interactions with nitrogen fixing rhizobia [27,28]. *Anthyllis vulneraria* L. (kidney vetch) is one of the dominant legume species on calamine waste deposits in Bolesław [1,8,9]. This species can grow in soils contaminated with TMs as well as in unpolluted soils, which indicates its great adaptability [2,3,4,8,9]. Studies on kidney vetch revealed divergent data regarding its ability to accumulate trace metals like Zn, Pb or Cd. Some of them indicated roots as the main sites of metal accumulation [27], whereas other showed metal accumulation, especially Zn, in aerial parts of the plants [1,29].

Our previous study [8] showed that roots and nodules of calamine *Anthyllis* ecotype implemented an avoidance strategy involving cell wall thickening in the presence of TMs. However, such studies are not available for leaves. We hypothesized that TMs may lead to (i) leaf apoplast modifications and (ii) changes in the antioxidant machinery efficiency to facilitate plant growth under severe contamination. To verify our hypothesis, we implemented immunolabelling, transmission electron microscopy and biochemical measurements. We also investigated metal accumulation strategy of the species. 

## 2. Materials and Methods

### 2.1. Plants Collection

Plants of metallicolous *Anthyllis vulneraria* L. ecotype (referred to as M) were collected in July 2017 on a 100 years old calamine heap in Bolesław near Olkusz, Ore-bearing Region (50°17′ N 19°29′ E), southern Poland, while plants of non-metallicolous ecotype (referred to as NM) inhabited a control site, i.e., a closed stonepit in Kazimierz Dolny (51°19′ N 21°56′ E). This place was chosen on the basis of similarity to the calamine environment conditions in terms of the solar exposure, soil permeability and alkaline pH [30]. 

### 2.2. Determination of Trace Metal Content in the Substrate and Plant Tissues

The rhizosphere soil samples of *A. vulneraria* M and NM ecotypes were taken from the calamine and control soil surface at sites where this species dominated in the plant cover from three sample points. At least three specimens were collected from each point for obtaining a representative sample. These samples consisted of both the plants, being at the flowering phase, and the soil bulk, containing the roots and the soil of the rooting zone (10–20 cm depth). The soil samples were air-dried, sieved through a 2-mm mesh and stored at room temperature until analysis. The plants were washed carefully with deionized water and dried to a constant mass. The samples were sent to Bureau Veritas Mineral Laboratories (Canada) for plasma mass spectrometry (ICP-MS) analysis.

The content of metals was determined in both shoots and roots of M and NM plants. For each metal, the translocation factor (TF) was calculated as follows: TF = metal content in the shoots (mg kg^−1^ DW)/metal content in the roots (mg kg^−1^ DW). 

Histochemical localization of Zn, Pb and Cd ions in plant tissues was performed with dithizone (diphenylthiocarbazone) (Sigma-Aldrich, St. Louis, MO, USA), which forms red complexes with metal ions [31]. Randomly chosen leaves were soaked for 30 min in the staining solution containing 30 mg dithizone, 60 mL acetone, 20 mL deionized water and a few drops of 45% (v/v) acetic acid. After washing with deionized water, free-hand leaf sections were prepared and observed under a light microscope AX70 Provis (Olympus Poland, Warsaw, Poland).

### 2.3. Leaf Structural Examination

For anatomical observations, ten fragments (approximately 5 × 2 mm) of fully expanded leaves randomly collected from M and NM plants were fixed in 2% (v/v) paraformaldehyde (Sigma-Aldrich, St. Louis, MO, USA), and 2% (v/v) glutaraldehyde (Sigma-Aldrich), in 0.1 M sodium cacodylate buffer (Sigma-Aldrich) (pH 7.2) for 2 h, rinsed four times with 0.1 M sodium cacodylate buffer and post-fixed in 1% osmium tetroxide for 3 h at 4 °C. Then, samples were dehydrated in an ethanol series supplemented with propylene oxide and embedded in epoxy resin (EPON, Fluka, Buchs, Switzerland). After resin polymerization at 65 °C for 24 h, blocs were cut on semi-thin sections (3 µm) and stained with 1% toluidine blue prior to examination under a light microscope Olympus AX70 Provis (Olympus Poland, Warsaw, Poland). The measurements involved three leaves of each ecotype. The total thickness, number and thickness of palisade and spongy cell layers, size of parenchyma cells, epidermis thickness and stomata number per 600 μm of the leaf surface were measured using Olympus-Provis Cell Sens Standard program under 10 × magnification.

Ultrastructural analysis involved ten leaf samples of fully expanded leaves, randomly collected from M and NM plants. Ultra-thin (80 nm thick) leaf sections were taken from epoxy resin-embedded samples using a Leica UCT ultramicrotome (Leica Microsystems, Nussloch, Germany), were mounted on formvar-coated nickel grids and stained with uranyl acetate, followed by lead citrate (Sigma-Aldrich) for 1 min and examination under an FEI 268D ‘Morgagni’ (FEI Comp., Hillsboro, OR, USA) transmission electron microscope (TEM) equipped with a 10 MPix Olympus-SIS ‘Morada’ digital camera (Olympus-SIS, Münster, Germany). The images were saved as jpg files and if necessary adjusted using Photoshop CS 8.0 (Adobe Systems, San Francisco, USA) software by non-destructive tools (levels and/or contrast) throughout the whole area of an image. The figures were prepared using Corel DRAW 11 software (Corel Corporation, Ottawa, Canada).

### 2.4. Cell Wall Component Immunolocalization

For fluorescent microscopy, leaf fragments were fixed in 4% paraformaldehyde in microtubule stabilizing buffer (MSB; 0.1 M Pipes, pH 6.8, 1 mM EGTA, 0.5 mM MgCI_2_) (Sigma-Aldrich) for 2 h at room temperature. Afterwards, samples were washed with MSB buffer, dehydrated in graded ethanol series and embedded in butyl-methacrylate (BMM) (Sigma-Aldrich) resin. After resin polymerization under UV at −22 °C for 24 h blocs, were cut on semi-thin (3 µm) sections and were placed in a drop of water on silane slides. After resin removal with acetone, further immunofluorescence procedure was carried out as described by Sujkowska-Rybkowska and Borucki [32]. To localize non-cellulose wall components we used sets of primary rat monoclonal antibodies from Plant Probes (Leeds, UK): Lm25 for galactosylated xyloglucan, Lm19 directed against low methyl-esterified homogalacturonan, Lm20 for highly methyl-esterified-homogalacturonan, Lm13 for (1-5)-α-L-arabinan, Lm2 for arabinogalactan AGP protein and Jim12 for extension. As a secondary antibody, we used goat anti-rat IgG conjugated with FITC (fluorescein isothiocyanate) (Sigma-Aldrich), and control reactions were performed without the primary antibody. Following immunolabelling the sections were post-stained with 0.1% toluidine blue (pH 5.5, 0.2 M sodium phosphate buffer) for 10 min to minimize tissue autofluorescence, and examined under a fluorescent microscope (AX70 Provis, Olympus Poland, Warsaw, Poland) equipped with UM61002 filter set and Olympus UC90 HD camera. For images in a bright field, equivalent sections were stained with 1% toluidine blue (Sigma-Aldrich) for 5 min at room temperature and then washed with distilled water. The images were collected from three independent experiments. For each, antibody pictures were taken for six fields of vision. 

For immunogold labelling leaf fragments were fixed in a medium containing 2% paraformaldehyde (Sigma-Aldrich), 2% glutaraldehyde (Sigma-Aldrich) in 0.1 M phosphate-buffered saline (PBS) (Sigma-Aldrich) pH 7.6 for 2 h at room temperature and embedded in Epoxy (Fluka, Buchs, Switzerland) resin. Then, 50–70nm thick sections were mounted on formvar-coated nickel grids and treated with 5% hydrogen peroxide (Sigma-Aldrich) for 8 min to remove the resin. Then, the grids were blocked for 1 h with 3% bovine serum albumin (Sigma-Aldrich) in 0.1 M PBS, rinsed three times with PBS and treated with primary antibodies (as described above) in PBS overnight at 4 °C. After washing with PBS, the sections were treated for 90 min with 10 nm gold-conjugated goat secondary antibody (Sigma-Aldrich) and rinsed two times for 5 min with PBS and distilled water. Labelling specificity was checked by omitting the primary antibody during incubation. The grids with the leaf sections were examined by TEM (as described above). Digital images were saved as jpg files and if necessary adjusted in Photoshop CS 8.0 (Adobe Systems, USA) software by non-destructive tools (levels and/or contrast) throughout the whole area of the image. Figures were prepared using Corel DRAW 11 software (Corel Corporation, Canada).

### 2.5. Electrophoresis and Western Blotting 

For total protein extraction, 100 mg of leaf powder, obtained by the sample grinding in liquid nitrogen with mortar and pestle, were incubated for 10 min in 1 mL of extraction buffer (65 mM Tris-HCl, pH 6.8, 2% SDS, 2 mM EDTA and 700 mM *β*-mercaptoethanol) (Sigma-Aldrich) and then vigorously vortexed, boiled in a water bath for 2 min, cooled on ice and centrifuged (4 °C, 2 min, 14,000× *g*). Protein content was determined using bovine serum albumin (Sigma-Aldrich) as a standard and 20 μg of protein were loaded into 10% SDS-PAGE gel with 4% stacking gel, with 14, 000–66,000 Molecular Weight Marker (Sigma-Aldrich). Electrophoresis was performed in 0.025 M Tris/0.192 M glycine/0.1% SDS buffer pH 8.3 at 160 V (for two gels) for 2 h (Mini-Protean electrophoresis system; Bio-Rad, Hercules, CA, USA). Proteins were blotted on polyvinylidene difluoride membrane using a buffer containing 25 mM Tris-HCl, 192 mM glycine, 10% methanol (Sigma-Aldrich) in a transfer pack (BioRad Trans-blot Turbo Transfer, Bio-Rad, Hercules, CA, USA) for 30 min. After the transfer, the blots were incubated for 1 h at room temperature in 5% powdered skimmed milk in Tris buffered saline (TBS) (10 mM Tris-HCl, 150 mM NaCl, pH 7.6) (Sigma-Aldrich) and washed three times for 10 min in TBS. Subsequently, the membrane was incubated overnight at 4 °C with primary rat monoclonal antibody (Jim12 anti-EXT or Lm2 anti-AGP) (Plant Probes, Leeds, UK) all diluted 1:1000 in 2.5% skimmed milk in TBS. Afterwards, the membranes were washed three times for 5 min in TBS and incubated for 90 min at room temperature (RT) with alkaline phosphatase-conjugated goat anti-rat antibody (Sigma-Aldrich) diluted 1:10 000 in TBS and then washed three times for 5 min in TBS. Next, the blots were mounted in an alkaline phosphatase detection buffer (100 mM Tris-HCl, 100 mM NaCl, 5 mM MgCl_2_, pH 9.5; Sigma-Aldrich) containing the substrate (6.6 µL/mL NBT and 3.3 µL/mL bromo-chloroindolyl-phosphate; Sigma-Aldrich). The reaction was stopped by rinsing with distilled water. Images were made in a Bio-Rad ChemiDoc Universal Hood II Gel Documentation System (Bio-Rad, Hercules, CA, USA) using Image Lab 5.2 software. For each blot, two technical repetitions were performed.

### 2.6. Photosynthetic Pigment Content Estimation

One hundred milligrams of leaf samples were homogenized with 80% acetone supplemented with CaCO_3_ and centrifuged (4 °C, 15 min, 4800× *g*). The extract absorbance for chlorophyll *a* (chl *a*), chlorophyll *b* (chl *b*) and carotenoids (car) was measured at 470, 646, and 663 nm, respectively, in a Nunc U-bottom 96-well plate (Thermo Scientific, Waltham, MA, USA) on a Varioskan LUX Multimode Microplate Reader (Thermo Scientific, Waltham, MA, USA). The pigment contents were calculated according to Wellburn [33] equations and expressed as mg of FW of the sample. Additionally, the content of total chlorophylls (chl *a* + *b*) as well as chlorophyll *a* to *b* ratio (chl *a*/*b*) were calculated.

### 2.7. Phenolic Compounds 

Total phenols, phenylpropanoids, flavonols and anthocyanins were determined according to Fukumoto and Mazza [34]. One hundred mg of leaf samples were homogenized with 80% methanol and centrifuged (4 °C, 15 min, 4800× *g*). Then, extracts were mixed with 0.1% HCl (in 96% ethanol) and 2% HCl (in water) and after 15 min the absorbance at 280, 320, 360 and 520 nm was read in UV-Star 96-well plate (Greiner, Monroe, NC, USA) on a Varioskan LUX Multimode Microplate Reader. Chlorogenic acid, caffeic acid, quercetin and cyanidin were used as standards for determination of particular phenolic groups. The content of phenolic compounds was expressed in mg of the respective standard equivalents per 100 g of FW. 

### 2.8. Radical Scavenging Activity (RSA)

The ability of methanolic extracts to quench a stable free radical 2,2-diphenyl-1-picrylhydrazyl (DPPH) (Sigma-Aldrich) was used to determine the radical scavenging activity of *Anthyllis* leaves [35]. Leaf extracts were mixed with DPPH solution, and absorbance was measured at 517 nm at the moment of reaction initiation and 30 min after the extract addition. The antioxidant activity of extracts was expressed in % of reduced DPPH· radical by a unit of plant extract.

### 2.9. Thiobarbituric Acid-Reactive-Substances (TBARs)

The thiobarbituric acid-reactive-substances (TBARs) amount, reflecting the level of lipid peroxidation, was estimated after homogenization of 100 mg of plant material with 80% methanol (Sigma-Aldrich). Samples were centrifuged (4 °C, 20 min, 16,000× *g*) and the methanolic extract was added to 0.5% 2-thiobarbituric acid dissolved in 20% trichloroacetic acid (Sigma-Aldrich). After incubation at 90 °C for 20 min, reactions were stopped in an ice bath. The samples were centrifuged (16,000× *g*, 10 min) and the absorbance was measured at 440, 532 and 600 nm in Nunc U-bottom 96-well plates on a Varioskan LUX Multimode Microplate Reader. TBARs level was calculated according to Hodges et al. [36] and expressed in µmol per gram of fresh weight (FW).

### 2.10. ROS Accumulation

The presence of O_2_^−^ in *Anthyllis* leaves was detected in situ with nitroblue tetrazolium (NBT). Leaves were immersed in a reaction mixture containing 0.2% (w/v) NBT, 2 mM dimethyl sulfoxide (DMSO) and 10 mM sodium azide in 50 mM sodium phosphate buffer (pH 7.5) (Sigma-Aldrich), and incubated in the dark for 3 h at room temperature. Superoxide reacts with NBT to produce a blue insoluble formazan compound. Hydrogen peroxide was localized by infiltration of the leaves with diaminobenzidine (DAB) according to Salzer et al. [37], as H_2_O_2_ forms brown complexes with DAB. Afterwards, the leaves were placed in a clearing solution with acetic acid/glycerol/ethanol (1/1/3, v/v/v) mixture to remove chlorophylls [38]. 

Hydrogen peroxide was also localized cytochemically using a TEM technique with CeCl_3_ that reacts with H_2_O_2_ to produce electron-dense deposits of cerium perhydroxides [39]. The leaf samples were incubated in 5 mM CeCl_3_ (Sigma-Aldrich) in 50 mM MOPS (3-*N*-morpholino-propanesulfonic acid, pH 7.2) (Sigma-Aldrich) for 1 h at room temperature. Afterwards, they were fixed in 2% (v/v) paraformaldehyde (Sigma-Aldrich) and 2% (v/v) glutharaldehyde (Sigma-Aldrich) in 0.1 M sodium cacodylate buffer (pH 7.2) for 1 h, washed twice with 0.1 M cacodylate buffer and post-fixed in 1% (v/v) osmium tetroxide (Sigma-Aldrich) for 45 min. After dehydration the samples were embedded in epoxy resin (Fluka, Buchs, Switzerland) according to the classic method for TEM described above.

### 2.11. Antioxidant Enzymes 

Catalase (CAT) and peroxidase (POX) cytochemical localization was carried out in the leaves according to Frederick and Newcomb [40], and Olmos et al. [41] with some modifications. The leaves were fixed in 2.5 % (v/v) glutaraldehyde (Sigma-Aldrich) in 0.1 M sodium cacodylate buffer (Sigma-Aldrich) pH 7.2 for 1 h, and then rinsed four times in the same buffer. For CAT activity visualization, the leaf samples were pre-incubated in 0.05 M Tris-HCl buffer (pH 9.0) (Sigma-Aldrich) for 30 min at 37 °C and then incubated for 1 h in a medium containing 2 mg/mL 3,3′-diamino-benzidine (DAB) (Sigma-Aldrich) and 0.06 % (v/v) H_2_O_2_ (Sigma-Aldrich) in 0.05 M Tris-HCl buffer (pH 9.0). Afterwards, the samples were washed four times in 0.05 M K/Na-phosphate buffer (pH 6.8). As a control reaction confirming CAT specificity, the samples were incubated in DAB medium without H_2_O_2_.

For POX localization, leaf samples were pre-incubated for 30 min at 37 °C in 0.05 M Tris-HCl buffer (pH 7.6) with 0.02 M 3-amino-1,2,4-triazole (CAT inhibitor) (Sigma-Aldrich). Next, the samples were incubated in a medium containing 0.5 mg/mL DAB (Sigma-Aldrich), 0.01% (v/v) H_2_O_2_ (Sigma-Aldrich), 0.02 M 3-amino-1,2,4-triazole in 0.05 M Tris-HCl (pH 7.6) for 1h at room temperature. Control leaf samples were incubated in DAB medium without H_2_O_2_. Afterwards, all samples were washed four times with 0.05 M K/Na-phosphate buffer (pH 6.8). The next steps were the same for CAT and POX localization. The samples were post-fixed in 1% osmium tetroxide (Sigma-Aldrich) in 0.1 M sodium cacodylate buffer (pH 7.2) for 2 h at 4 °C. Then, they were dehydrated through an ethanol series and embedded in Epoxy resin (Fluka, Buchs, Switzerland) according to the manufacturer’s procedure. Next, the resin blocks were cut into 3 µm thick sections and observed under a light microscope Olympus AX70 Provis (Olympus Poland, Warsaw, Poland).

For measuring enzymes’ activity, leaf samples consisting of 100 mg of plant material were frozen in liquid nitrogen and then stored at −80 °C until protein extract preparation. Tissue homogenates were obtained by grinding of the samples in a mortar with quartz sand and ice cold extraction medium (pH 7.2) (50mM 3-(N-morpholino) propanesulfonic acid (MOPS), 2 mM 2-mercaptoethanol, 0.1 mM ethylenediaminetetra acetic acid (EDTA), 2% polyvinylpyrrolidone, 0.5% Triton X-100, 1 mM phenylmethylsulfonyl fluoride (Sigma-Aldrich)). The samples were incubated on ice for 20 min and centrifuged (4 °C, 20 min 16,000× *g*). The extracts were stored at −80 °C until analysis. 

Superoxide dismutase (SOD) activity was measured according to Kostyuk and Potapovich [42]. A reaction medium was obtained by mixing equal aliquots of 67 mM K/Na phosphate buffer (pH 7.8) and 25 mM EDTA (Sigma-Aldrich), the pH value of this solution was adjusted to 10.0 with tetramethylethylenediamine (Sigma-Aldrich). The extract was mixed with the reaction medium and 2.5 μM quercetin in dimethyl sulfoxide (Sigma-Aldrich) were added. Assays were conducted in Nunc U-bottom 96-well plates on a Varioskan LUX Multimode Microplate Reader. The absorbance at 406 nm was recorded for 20 min with one reading per minute. SOD activity was expressed in arbitrary units (the amount of SOD that inhibits superoxide-driven oxidation of quercetin by 50%) per gram of FW. 

For CAT activity measurement according to Aebi [43], the extract was mixed with 50 mM Tris-HCl buffer (pH 7.0) and 0.168 % H_2_O_2_ (Sigma-Aldrich) in the same buffer. The assays were performed at 37 °C in UV-Star 96-well plates on a Varioskan LUX Multimode Microplate Reader. The absorbance at 240 nm was recorded for 10 min with absorbance readings every 30 s. CAT activity was expressed as decomposition of µmol of H_2_O_2_ per minute and gram FW. 

For POX activity measurement according to Lück [44], the extract was mixed with an assay medium (0.49 % (w/v) *p*-phenylenediamine and 0.049 % (v/v) H_2_O_2_ in 50 mM Tris-HCl buffer, pH 6.8) (Sigma-Aldrich). The assays were performed at 37 °C in Nunc U-bottom 96-well plates on a Varioskan LUX Multimode Microplate Reader. The absorbance at 485 nm was recorded for 10 min with absorbance readings every 30 s. Oxidation of PPD by POX/H_2_O_2_ yielded Bandrowski’s base and POX activity was expressed in arbitrary units. One unit of POX activity was defined as a 0.1 increase of absorbance after 1 min per gram FW.

### 2.12. Statistical Analyses

All results were statistically analysed by one-way analysis of variance (STATISTICA ver. 13.3 TIBCO Software Inc., Palo Alto, USA), and significant differences were determined following a post-hoc Tukey’s test. 

## 3. Results

### 3.1. Trace Metal Content in the Soil and Plant Tissues

Total metal content in the calamine soil exceeded 10,000 mg kg^−1^ of Zn, 1560.90 mg kg^−1^ of Pb and 129.14 mg kg^−1^ of Cd. The control soil contained 55.6 mg kg^−1^of Zn, 10.4 mg kg^−1^ of Pb and 1 mg kg^−1^ of Cd. 

The plasma mass spectrometry analysis shows that the metallicolous (M) shoots accumulated lower amounts of Zn, Pb and Cd than the roots but their content in particular organs reached statistically higher levels compared to non-metallicolous (NM) plants. In the shoots of M ecotype, Zn concentration amounted 2115.03 mg kg^−1^ dry weight (DW), whereas in the roots it was about two times higher (Table 1). Pb accumulation in M shoots was 75.88 mg kg^−1^ DW, while in the roots Pb content was three times higher. The metallicolous ecotype shoots also contained a high amount of Cd (18.9 mg kg^−1^ DW), but again three times lower than the roots. Zn content in NM plants was relatively low and reached only 10 and 18 mg kg^−1^ DW in the shoots and roots, respectively. Similarly, Pb and Cd accumulation in M shoots was lower than in their roots, even though their uptake was significantly higher in M than NM plants. For NM plants it was 2.99 mg kg^−1^ DW of Pb and 0.25 mg kg^−1^ DW of Cd. Despite this, the translocation factor (TF), which expresses the plant’s ability to transfer metals from roots to shoots, was similar for NM and M ecotype (Table 1), and was below 1 for all the analysed metals, indicating metal retention in the roots.

**Figure 1:**
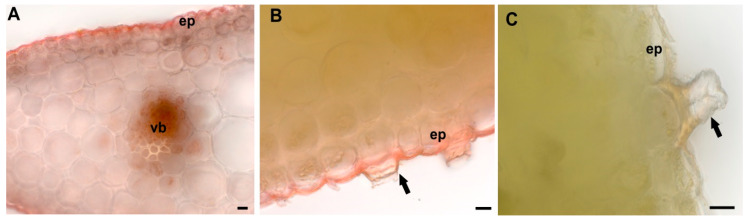
Dithizone staining of *Anthyllis* leaves for metal localization. (**A**,**B**) Metallicolous ecotype. Visible red metal-dithizonate complexes in veins (vb), epidermis (ep) and non-glandular trichomes fragments (arrow) of leaves. (**C**) Non-metallicolous ecotype leaf and no red colour is visible. Scale bars = 10 µm.

Metal localization in leaf tissues was visualized by staining with dithizone that formed metal-dithizonate complexes (Figure 1). The colour reaction was only visible in M leaves and occurring in veins, epidermal cell walls and trichomes.

### 3.2. Leaf Anatomical and Ultrastructural Studies 

**Figure 2:**
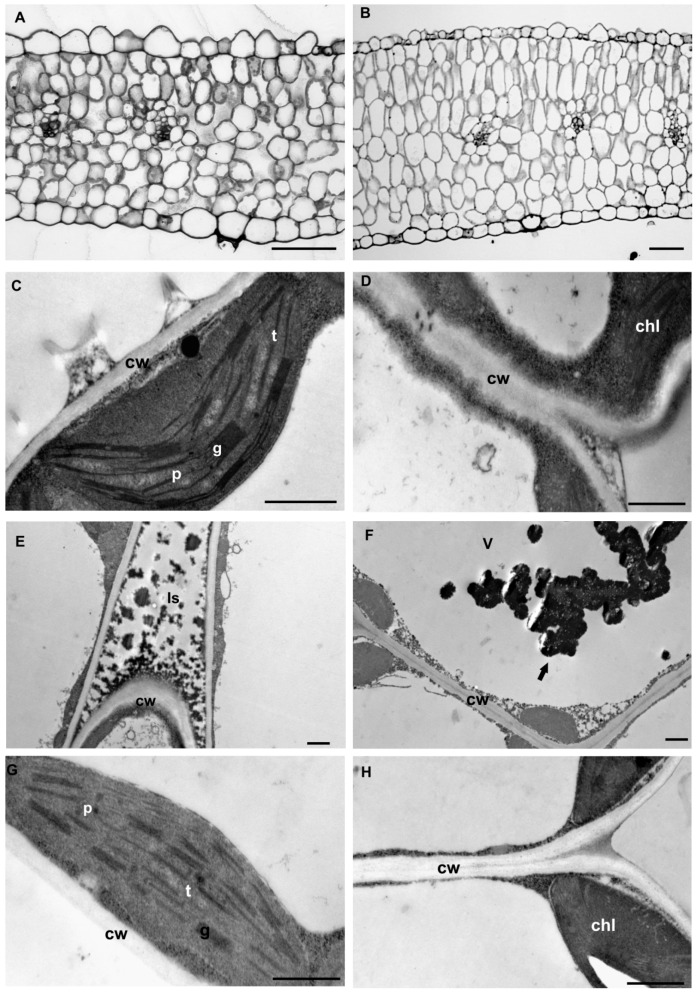
Light micrographs of leaf cross-sections of metallicolous (**A**) and non-metallicolous (**B**) *Anthyllis* ecotypes. Bars = 50 µm. Transmission electron micrographs (**C**–**H**) showing the ultrastructure of mesophyll cell from metallicolous (C–F) and non-metallicolous (G,H) *Anthyllis* leaves. (**C**) Visible large chloroplast with regular structure with expanded grana (g) and small plastoglobules (p). (**D**) Thick cell wall (cw) of mesophyll cell. (**E**) Dark precipitates in intercellular spaces (Is) and phenolic-like inclusions (arrow) in vacuole (**F**). (**G**) The regular structure of chloroplasts from NM plants. (**H**) Thin-walled mesophyll cell of NM leaves. chl: chloroplast; t: thylakoid; v: vacuole. Bars = 1 μm.

Analysed *Anthyllis* leaves showed a typical dorsiventral structure, with non-glandular trichomes at the abaxial leaf surface. The smallest and most narrow leaves were characteristic of M specimens, while the NM leaves were much thicker (Figure 2A,B). Their mesophyll consisted in palisade and spongy parenchyma. The ecotypes differed also in their anatomical features. 

The microscopic measurements revealed that the M ecotype had significantly thinner leaves than the NM one (206 vs. 273.7 μm for M and NM, respectively) (Table 2). Moreover, M leaves showed looser packing of mesophyll cells and a thinner spongy layer than NM leaves. Reduced leaf thickness in M plants resulted from a lower number of palisade and spongy layers. Additionally, the M ecotype had significantly shorter palisade and spongy cells as compared with the NM ecotype. Statistically significant differences also occurred in the thickness of the epidermis, which was thicker in M leaves. Moreover, in M leaves the number of stomata was twice lower than that in NM leaves and almost two times higher on the adaxial than on the abaxial surface. Contrary to that, the number of stomata in both epidermis layers of NM leaves was quite similar.

Transmission electron micrographs obtained from M and NM *Anthyllis* leaves showed differences in leaf ultrastructure (Figure 2C–H). Noticeable changes occurred for wall thickness and chloroplast size and thylakoid system organization. The mesophyll cells of M leaves were surrounded by thick walls (Figure 2D), and had well-differentiated large chloroplasts containing expanded grana and well-developed stroma lamellae with numerous stroma electron-translucent spaces connected with starch formation (Figure 2C). Additionally, the intercellular spaces and vacuoles of M leaf mesophyll contained dark precipitates (Figure 2E). The vacuoles of mesophyll cells harboured also phenolic-like inclusions (Figure 2F). Non-metallicolous mesophyll cells were thin-walled with smaller and less numerous chloroplasts containing a well-developed grana/intergranal thylakoid system, and a lighter stroma than M leaves (Figure 2G,H). 

### 3.3. Leaf Apoplast Alterations

**Figure 3:**
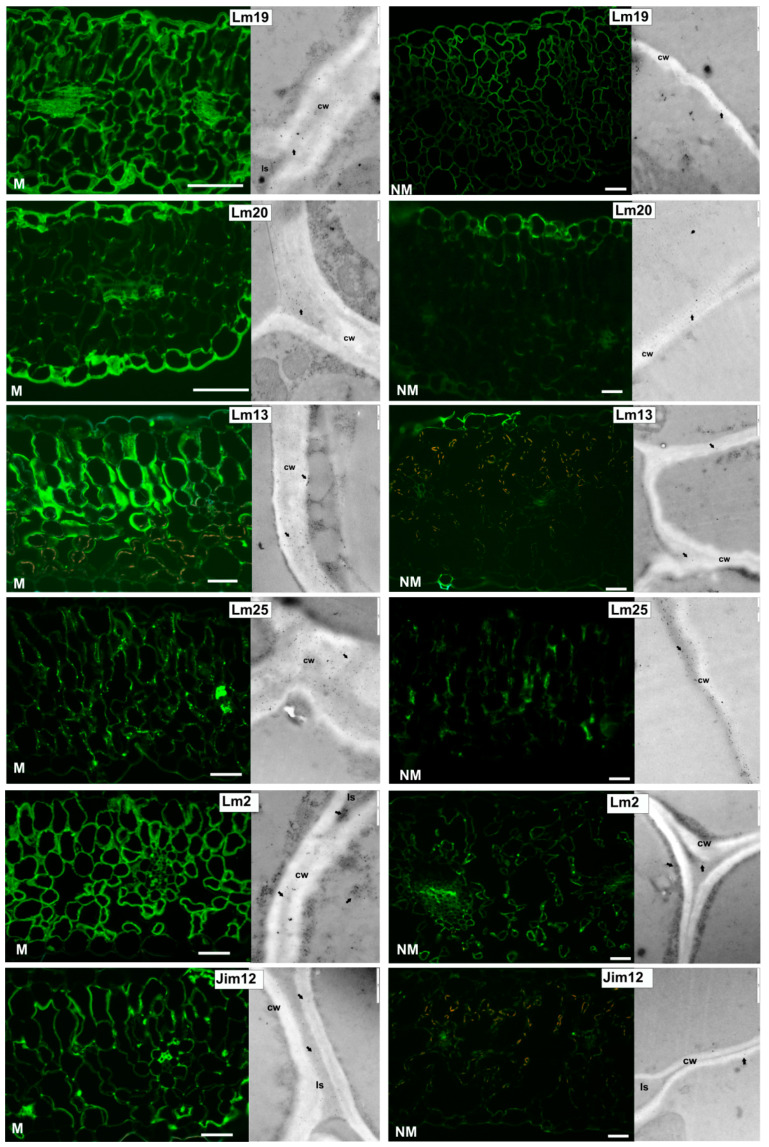
Localization of cell wall components in transversal sections of metallicolous (M) and non-metallicolous (NM) *Anthyllis* leaves using immunofluorescence and immunogold labelling. Note that the M leaves are strongly labelled with all antibodies. Arrows indicate colloidal gold deposits. cw:cell wall; Is:intercellular space. Bars = 50 μm.

Structural changes within the leaf apoplast of *Anthyllis* ecotypes were revealed by immunohistochemical studies using monoclonal antibodies that bind to selected pectins (Lm19 and Lm20), (1-5)-α-L-arabinan (Lm13), xyloglucan (Lm25), arabinogalactan protein (AGP) (Lm2) as well as extensin (EXT) (Jim12) (Figure 3). Immunofluorescent analysis with Lm2 antibody detected high level of AGP in M leaves, especially along the walls, and in the apoplastic space of mesophyll cells, while Lm2 was less visible in NM leaves (Figure 3). Jim12 against EXT showed a similar wall and intercellular space fluorescence labelling pattern in M and NM leaves, with an overall signal stronger in M ecotype. Immunogold labelling confirmed immunofluorescence results for Lm2 and Jim12. Numerous gold particles of Lm2 were observed in the cytoplasmic compartments, cell wall and intercellular spaces of M leaves, while gold staining in NM cells was less intense but occurred in the same locations. For EXT detection, intense immunogold labelling was observed in the cell walls and intercellular spaces of M leaves, while in NM leaves the labelling was less significant. 

The presence of pectic homogalacturonan (HG), a major pectic polymer, was visualized using Lm19 and Lm20 antibodies (Figure 3) towards low/non methyl-esterified HG and highly methyl-esterified HG, respectively. Immunofluorescent study showed that M leaves, contrary to NM ones, were abundant in both de-esterified and heavily methyl-esterified pectins. A strong fluorescent signal was located in the thick epidermis of M leaves. Lm19 epitopes were abundant in all cell walls and intercellular spaces of M leaves, whereas Lm20 was present in the epidermis and mesophyll intercellular spaces. Intense gold labelling of Lm19 was observed under the electron microscope in the intercellular spaces and cell walls, especially in the middle lamella. Gold granules of Lm20 epitopes were present in the epidermis and intercellular spaces in M leaves, while NM cells showed less intense gold labelling. 

Immunofluorescent analysis recognizing pectic arabinan shows strikingly different binding patterns of Lm13 in *Anthyllis* leaves (Figure 3). Lm13 arabinan epitopes were abundant in M leaves in the epidermis and palisade parenchyma walls, whereas their presence in NM leaves was barely detectable in the epidermis and veins (Figure 3). Gold particles were numerous in the walls of M leaves, and sparse in the walls of NM leaves (Figure 3).

Immunofluorescence assay with Lm25 antibody showing the distribution of xyloglucan, the major non-cellulosic polysaccharide in dicots, revealed a stronger presence of xyloglucan in the cell walls of M than NM leaves (Figure 3). Immunogold results corroborated the immunofluorescence outcomes. Thick walls of M leaves show numerous grains of gold. 

**Figure 4:**
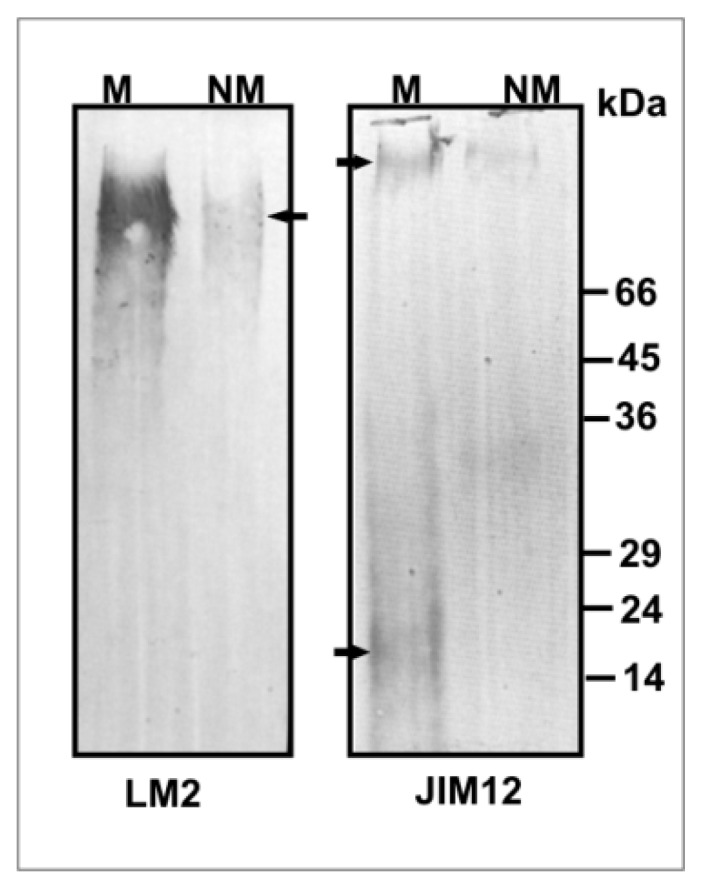
Immunoblot analysis of arabinogalactan protein (Lm2) and extensin (Jim12) antigens in extracts of metallicolous (M) and non-metallicolous (NM) *Anthyllis* leaves. Aliquot containing 20 μg of proteins was loaded per lane. The position of the molecular weight marker is indicated.

To learn more about the actual amount of structural proteins-AGP and EXT in the leaves, a Western blot analysis was performed (Figure 4). Western blot assay confirmed immunofluorescence and immunogold labelling indicating higher AGP and EXT accumulation in M leaves vs. NM ones. Lm2 antibody recognized a smear fraction at the top of the resolving gel (above 70–100 kDa), predominantly for the M ecotype. Jim12 antibody recognized a fraction of molecular weight exceeding 100 kDa in NM, whereas in M leaves this antibody detected glycoproteins of 100 kDa and 20 kDa, which were not present in NM tissues.

### 3.4. ROS Accumulation 

**Figure 5:**
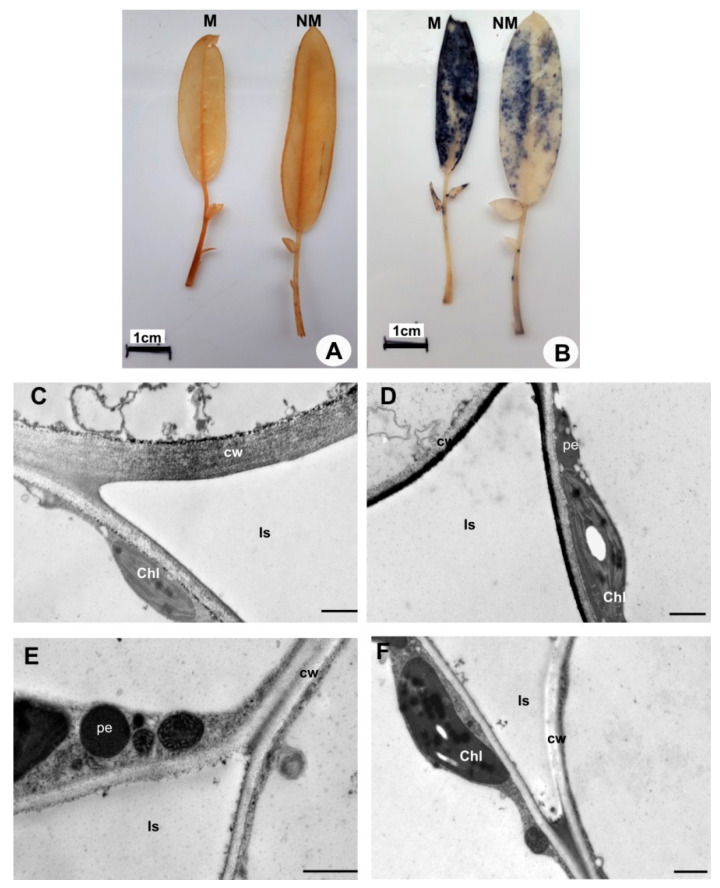
Localization of reactive oxygen species. Visualization of hydrogen peroxide (H_2_O_2_) molecules by diaminobenzidine staining (**A**) and localization of superoxide anions using nitrotetrazolium blue chloride (**B**) in detached leaves. Bars = 1 cm. (**C**–**F**). Cytochemical localization of H_2_O_2_ in mesophyll cells of metallicolous M (**C**,**D**) and non-metallicolous NM (**E**,**F**) leaves. Electron-dense deposits of cerium perhydroxides indicate H_2_O_2_ localization. Chl: chloroplasts; cw: cell walls; Is-intercellular spaces; pe: peroxisome. Bars = 1 µm.

ROS accumulation differed between *Anthyllis* leaves (Figure 5). For in situ H_2_O_2_ localization diaminobenzidine (DAB) yielding a brown polymerization product was used. DAB staining of leaf blades was similarly strong in both ecotypes, however, petioles showed greater peroxide accumulation in M leaves (Figure 5A). In a reaction with nitrotetrazolium blue chloride (NBT) detecting superoxide anions, M leaves showed strong blue coloration of the entire leaf blade (Figure 5B). In NM leaves, the localization of superoxide was similar but much less intense.

Hydrogen peroxide localization in *Anthyllis* leaf blade was confirmed by subcellular reaction with CeCl_3_ and TEM analysis (Figure 5C–F). In M leaves, the main site of H_2_O_2_ accumulation was the apoplast (thick walls and intercellular spaces), and some of cerium perhydroxide deposits were also observed in the chloroplasts of mesophyll cells (Figure 5C,D). In NM leaves, cerium precipitates in the cell walls were less frequent, whereas the chloroplasts showed similar H_2_O_2_ accumulation (Figure 5E,F).

### 3.5. Activity of ROS Detoxifying Enzymes

**Figure 6:**
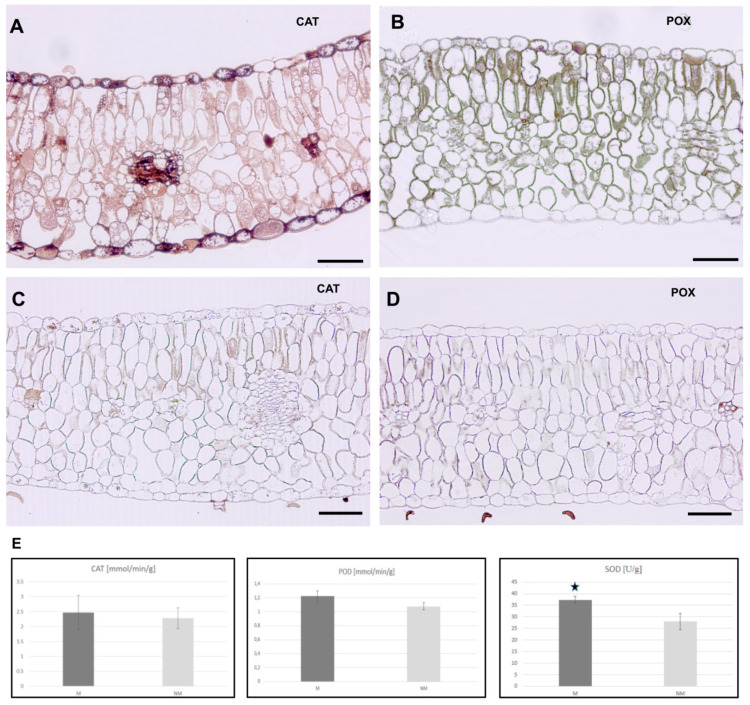
Antioxidant activity in leaves of *A. vulneraria* specimens. (**A**–**D**) Localization of catalase (CAT) and peroxidase (POX) activity in metallicolous (M) (**A**,**B**) and non-metallicolous (NM) (**C**,**D**) leaves. Dark brown coloration indicated enzyme presence. Bars = 50 μm. (**E**) The level of antioxidant enzymes’ activity in *Anthyllis* leaves. Asterisk indicates the significant difference at *p* < 0.05 according to one-way ANOVA and post-hoc Tukey’s test.

Histochemical detection of peroxisomal catalase (CAT) and peroxidase (POX) in the leaves shows differences between *Anthyllis* ecotypes (Figure 6). In M leaves, brown coloration indicating CAT presence was visible in the leaf epidermis, palisade and spongy parenchyma as well as in vascular bundles, while POX was visible in the upper epidermis and parenchymal cell walls (Figure 6A,B). In NM leaves, CAT was detected fragmentarily in both epidermis and weakly in leaf parenchyma, while POX was present only in mesophyll parenchyma and vascular bundles (Figure 6C,D). To learn more about the antioxidant enzymatic defence mechanisms, the activity of SOD, CAT and POX was measured spectrophotometrically. The readings demonstrate that CAT and POX activity did not differ significantly between M and NM plants but the activity of SOD was significantly higher (about 1.3 times) in M than NM plants (Figure 6E).

### 3.6. Plant Vitality-Physiological Measurements 

Plant physiological status was evaluated on the basis of photosynthetic pigment content in the leaves. *Anthyllis* ecotypes differed significantly when particular pigments were taken into consideration (Table 3). Plants of M population accumulated about 40% more chlorophyll *a* and *b* than NM ones, in which total chlorophyll content slightly exceeded 1 mg·g^−1^ fresh weight (FW). Despite this, chlorophyll *a* to *b* ratio was statistically similar in NM and M leaves and ranged from 3.1 to 3.4. Furthermore, the content of carotenoids in NM plants was lower by one-third in comparison with M ones and reached 0.15 mg g^−1^ FW.

As for phenolic compounds, the leaves of M ecotype accumulated remarkably higher amounts of their particular groups than NM ones (Table 3). In NM plants, the content of total phenols, phenylpropanoids and flavonols significantly decreased to almost 65%–66% that of M plants and reached 850, 250 and 340 mg g^−1^ FW, respectively. The greatest difference between the ecotypes was noticed for anthocyanins, a reduction of which (to 53 mg g^−1^ FW) was observed in NM leaves, whereas in M leaves their content was over three times higher. However, regardless of the ecotype, leaf extracts exhibited similar capacity to reduce DPPH radical at the level of 20% (Table 3). 

Plants representing the metal-polluted and control sites were also compared for their content of thiobarbituric acid-reactive-substances (TBARs) that indicate lipid peroxidation, and thus reflect the intensity of oxidative stress. Our analysis reveals statistically higher about 12 µmol g^−1^ FW concentration of TBARs in M leaves than in NM leaves, in which it reached almost 61 µmol g^−1^ FW (Table 3). 

## 4. Discussion

### 4.1. Zn, Cd, and Pb Contents in the Soil and Their Accumulation in Plant Tissues

Legumes spontaneously colonizing old calamine wastes are unique because they can tolerate huge amounts of toxic metals. In our experiment, mining wastes from which plants for analyses were harvested were proved to be highly contaminated with Zn, Pb and Cd. The metal occurrence exceeded many times the concentrations established by Polish law for industrial areas, i.e., 15,600 and 1000 mg kg^−1^ for Cd, Pb, and Zn respectively [45]. 

Our study shows that similarly to other legume plants growing on metal contaminated sites [46], *Anthyllis* M ecotype accumulated Zn, Pb and Cd mainly in the roots. Metal immobilization in the roots is a defence strategy common for metal-tolerant plants [47,48]. Although TMs accumulated by M plants are mostly retained in the roots, significant amounts reach the aerial parts. Normally, root endodermis with Casparian strip should limit the apoplastic metal ion transport to the shoots [49,50]. However, at high TMs content in the soil, the plant mechanisms limiting the mobility of toxic ions are overcome and some TMs may enter the aerial parts by passing the endodermal barrier or through the root tips lacking a well-developed Casparian strip [51]. In our experiment, M *Anthyllis* shoots accumulated significant amount of Zn and Pb that exceeded the toxicity level in plants [52]. Taking these metal levels into account, *A. vulneraria* calamine ecotype should be considered as a Zn-tolerant plant rather than a Zn hyperaccumulator, as Zn concentrations in its aerial tissues were below the hyperaccumulation threshold (levels above 3000 mg kg^−1^) [53]. This finding is in accordance with other researchers, who obtained similar results for *Anthyllis* plants growing on metal contaminated sites [1,3]. In our study, the presence of TMs in the leaves of metal-treated *Anthyllis* plants is also confirmed by histochemical staining with dithizone. Microscopic observations identify the epidermal cell walls, trichomes and leaf veins as the main sites of metal accumulation/translocation. This may suggest the way of metal detoxification and sequestration as reported for other metal-tolerant plants [17,52,53]. 

### 4.2. Leaf Anatomy

Even though TM content in M shoots exceeded permissible values, the plants showed no toxicity symptoms. Light microscope analysis revealed that the leaves of metallicolous plants were smaller, thinner and had fewer palisade parenchyma layers than those of non-metal tolerant plants. Plant adaptation to different environmental conditions is often associated with changes in the leaf structure at morphological and anatomical levels [11,54]. Plants growing on calamine wastes have to face not only trace metal contamination but also high insolation and drought [1]. Trace metal exposure reduces the size of mesophyll cells and leaf thickness [55]. The thinner palisade layer of metallicolous *Anthyllis* is probably compensated by the larger dimensions of the chloroplasts observed in the mesophyll cells. Additionally, thickened adaxial and abaxial epidermis with lower stomata number found in M specimens may be another strategy to minimize water loss. It was shown that adaxial and abaxial epidermis thickening and leaf thickness reduction often occur under metal stress [56]. Stomata presence mainly in the abaxial epidermis is an advantageous trait in plants growing in high temperature environments, as it could reduce the transpiration [47,54]. Moreover, lower transpiration rates may reduce trace metal uptake by plants [56]. The leaves of plants representing the metal-polluted site also show looser packing of mesophyll cells with large intercellular spaces, which may compensate for limited supply of CO_2_ for photosynthesis as observed in other metal-tolerant plants, e.g., *Brachiariade cumbens* [56] or *Silene vulgaris* [11]. This may also be the adaptation strategy of *Anthyllis* plants to stress conditions.

### 4.3. Leaf Apoplast Modifications

Plants spontaneously colonizing calamine wastes had to develop different adaptations to grow in these harsh conditions [57]. In our experiment, TEM analysis revealed that TMs exposure induced wall-thickening in the epidermis and mesophyll cells of M plants. Comparable increases in cell wall thickness were reported in shoots of *Vicia faba* exposed to TMs [58]. A similar apoplast modification was observed in our previous study in the roots and nodules of calamine *Anthyllis* ecotype [8]. Immunolabelling assays reveal that the apoplast of metallicolous leaves is rich in xyloglucan (labelled with Lm25 antibody), de-esterified and esterified pectins (Lm19 and Lm20 antibodies respectively), pectic arabinan (antibody Lm13), arabinogalactan protein (AGP, antibody Lm2) and extensin (EXT, antibody Jim12). These structural modifications of the apoplast under metal stress were observed in the leaves of this species for the first time. To cope with metal stress, plants alter their cell wall structure that forms a physical barrier against the metal entry into the symplastic compartment [12,23]. Trace metals triggered thickening of the epidermis and mesophyll walls in *Anthyllis* M leaves could be related to higher accumulation of polysaccharides-xyloglucan and pectins. According to Liepman et al. [58], cell wall thickening is associated with massive deposition of cellulose and hemicelluloses inside the primary cell walls. Metallicolous leaf apoplast was rich in low-methyl esterified pectins that may have resulted in cell wall stiffening and thickening. The degree of pectin methylesterification seems to determine TMs sequestration. Poorly methylesterified pectins, recognized by Lm19 antibodies and containing some amounts of free carboxyl groups, can efficiently retain divalent and trivalent metal ions [59,60]. It was shown that enhanced content of de-esterified pectin at the cell junctions of flax hypocotyls improved tissue cohesion and Cd sequestration [61]. Elongating cells with plastic cell walls featured high content of methyl-esterified pectins (Lm20) [62], whereas the maturing, stiffer walls were abundant in low methylesterified pectins (Lm19) [56]. Literature abounds in reports showing metal-triggered enhancement in cellulose, hemicellulose and pectin contents in plant roots and metal immobilization by cell wall polysaccharides as the main mechanisms of metal stress avoidance [12,22,23]. Binding metals leads to thickening and stiffening of the cell walls [22]. According to Gomes et al. [56], the deposition of trace metals in non-photosynthetic tissues like the apoplast could be a common plant strategy to tolerate toxic levels of TMs. 

Other cell wall compounds, such as structural proteins, can modify wall structure and also play protective role against toxic ions [23,24]. In this study, Western blot analysis and immunolabelling confirm high amounts of AGP and EXT in M leaves. AGP was prominent in thick walls and cytoplasmic compartments of metal-treated leaves, whereas EXT dominated in the walls and intercellular spaces. These findings indicate that the adaptation of *Anthyllis* leaves to trace metals is tightly regulated not only by increased levels of cell wall polysaccharides but also of wall structural proteins, like AGP and EXT. The genes encoding AGP and EXT are induced in response to abiotic stress [63]. Arabinogalactan proteins are highly glycosylated proteoglycans of the wall and plasma membrane, also present in cytoplasmic compartments [64,65]. These proteins secreted into the wall are abundant in negatively charged O-glycans and bind to positively charged pectins and hemicelluloses of the wall [66]. Arabinogalactan proteins play a signalling role at the cell surface, as they mediate signal transduction via the cell wall-plasma membrane-cytoskeleton continuum [67]. Some AGPs take part in secondary cell wall thickening [65]. It was shown that plasma membrane localized AGPs cross-linking to themselves in the presence of hydrogen peroxide support the cell wall integrity [68], similarly as with wall EXT [65,69]. Extensins are structural proteins induced by different stresses. Under TM stress they can bind metals and form a rigid insoluble matrix [70]. Cell wall oxidative cross-linking leads to the wall stiffening and can be catalysed by POX that uses H_2_O_2_ [71]. Oxidative coupling of tyrosine residues of EXT stiffens the wall structure enhancing its mechanical fortification [72]. This may be supported by the fact that POX was mainly active in the epidermal layers and mesophyll cell walls of M leaves. This correlated with a strong peroxide presence in the apoplast, suggesting the existence of mechanisms protecting mesophyll tissues from toxic TMs. Additionally, TMs presence in the epidermis wall was confirmed by dithizone staining. Moreover, EXTs form covalent bonds with pectins [73], but specific moieties involved in these linkages have not been identified yet. EXT in thickened cell walls and the intercellular spaces of metal treated M leaves may reduce metal migration and stiffen the walls. Our results show that in the leaves of calamine *Anthyllis* ecotype, TM detoxifying strategies consist mainly of apoplast remodelling due to the higher capacity of thick cell walls of TM immobilization. To conclude, our results suggest that in these leaves wall-related adaptation mechanisms may prevent metal translocation to the cytoplasm of photosynthetic tissues and their damage. 

### 4.4. Plant Vitality 

The effectiveness of the mechanical barrier limiting the toxic ion penetration into mesophyll cells is confirmed by TEM analysis, which shows no disintegration of cell organelles or membranes in M leaves. The chloroplasts of M ecotype contained expanded grana with small number of plastoglobuli that indicated intensive and undisturbed photosynthesis [74]. This was accompanied by high content of chlorophyll (*chl a* and *chl b*), the molecules of which are the main components of the grana. Increased content of *chl a* may result from low oxidative damage to chloroplast lipids, pigments and proteins [75]. Efficiency of the photosynthetic apparatus is undisturbed in metal-treated leaves, as reflected by similar chl *a*/*b* ratio in M and NM leaves. Additionally, metallicolous plants show increased carotenoid concentration, which could be an important sign of tolerance. As accessory photosynthetic pigments, carotenoids together with high chlorophyll levels are capable of maintaining photosynthetic activity [76]. On the other hand, they participate in antioxidant defence under metal stress [77,78]. Carotenoids bound in chloroplast membranes in lipid–protein complexes stabilize membrane lipids and protect membrane surface from oxidation [79]. *Anthyllis* plants representing the metal-polluted site had slightly elevated level of TBARs (lipid peroxidation indicator), as compared with controls, but the plants showed no signs of toxicity, thus indicating a lack of cellular oxidative stress. This way membrane-bound carotenoids can complement antioxidant system of metallicolous plants to counteract oxidative stress at the cellular level and maintain photosynthetic activity.

### 4.5. Activation of Antioxidant System under Metal Stress

An efficient antioxidant system prevents oxidative stress under TM exposure [11,79,80]. In our study, two components of the antioxidant system, SOD and phenols (especially anthocyanins), are particularly active in M ecotype of *Anthyllis* plants. They are deemed the main protectors against oxidative stress triggered by TMs presence. SOD is involved in the defence response by direct elimination of superoxide anion that is converted into hydrogen peroxide [81]. Increased SOD activity in our study may be explained by enhanced accumulation of superoxide that was visualized by NBT staining, whereas hydrogen peroxide was not so abundant in light microscope evaluation. Only TEM analysis confirms H_2_O_2_ localization in the apoplast of M leaves. It is highly probable that H_2_O_2_ bound to the cell walls is scavenged by POX to polymerize lignin and/or cross-link proteins and polysaccharides to improve tissue cohesion and metal immobilization [82]. Another mechanism engaged in both oxidative stress reduction and metal binding in *Anthyllis* leaves involves enhanced synthesis of phenols, especially anthocyanins. In general, phenolic compounds are often synthesized under metallic stress [11,83], since they can directly eliminate ROS, and thus inhibit lipid peroxidation of membranes by scavenging lipid alkoxyl radicals [84]. Moreover, anthocyanins, as plant vacuolar pigments belonging to flavonoids, are not only strong antioxidants [28,85] but also efficient chelators of TMs ions in these cellular compartments [86]. Taking into account that the vacuoles of mesophyll cells of *Anthyllis* plants representing the metal-polluted site harboured phenolic-like inclusions, it is highly probable that the lack of noticeable disturbances in metallicolous mesophyll cells results from metal immobilization in the vacuoles. In this way phenols, mainly vacuolar anthocyanins, could support the apoplast and limit metal presence in the symplast.

### 4.6. Symbiotic Associations

In addition to the structural adaptations and physiological mechanisms enabling *Anthyllis* plants to grow on calamine wastes, beneficial effects of their symbionts should also be considered. Our latest studies shows that *Anthyllis* plants are symbiotically active on old calamine wastes interacting with both bacteria (*rhizobia)* and arbuscular mycorrhizal fungi [8]. Rhizobia housed in root nodules provide the plants with reduced nitrogen, while arbuscular fungi supply phosphorus and water in exchange for photosynthetic products. Additionally, fungi hyphae have strong affinity for trace metals and limit TMs mobility [87]. Certainly, both these symbiotic systems require a lot of carbohydrates from the host, but the benefits that the plant receives in return outweigh the costs. These rhizospheric microorganisms play an important role in metal tolerance and alleviation of metal stress and increase plant survival at metal contaminated sites [87,88]. It is therefore highly probable that both *A. vulneraria* symbionts (fungi and bacteria) may further improve plant vitality on calamine wastes. However, this assumption needs further research. 

## 5. Conclusions

The results confirmed our hypotheses and indicate that: (A) *Anthyllis* plants from the metal polluted environment have an effective antioxidant system based on SOD activity and phenolic compounds; (B) thickened cell walls of metallicolous *Anthyllis* leaves may limit the metal ion movement and their toxic effects on the photosynthetic apparatus; (C) modified cell walls of metallicolous *Anthyllis* leaves contained enhanced amounts of hemicellulose, pectins and structural proteins. These cell wall-related adaptation mechanisms of calamine plants may curb the damage of mesophyll cells under toxic metal stress, play a potential role in metal stress avoidance and offer the plants the strength required for growth in metal polluted environments.

## Figures and Tables

**Table 1:** Content of Zn, Pb and Cd in kidney vetch shoots and roots (mg·kg^−1^DW) from calamine (M) wastes and control (NM) site.

M				NM		
	Shoots	Roots	TF	Shoots	Roots	TF
Zn	2115.03 a * ± 65.7	3857.90 a ± 137.84	0.5	10.10 b ± 1.04	18.87 b ± 2.16	0.5
Pb	75.88 a ± 20.74	252.68 a ± 32.45	0.3	1.23 b ± 0.57	2.99 b ± 0.28	0.4
Cd	18.90 a ± 5.10	59.50 a ± 0.81	0.3	0.08 b ± 0.01	0.25 b ± 0.02	0.3

TF—translocation factor. * Data present means ± SD. Statistical analysis was performed separately for shoots and roots; different letters indicate significantly different means at *p* < 0.05 according to one-way ANOVA and post-hoc Tukey’s test.

**Table 2:** Anatomical characteristics of leaves of different *Anthyllis* ecotypes.

Analysed Traits	M	NM
Total thickness (µm)	206.0 b * ± 22.38	273.7 a ± 38.25
Number of palisade cell layers	2	3
Thickness of palisade layer (µm)	65.1 b ± 8.03	102.9 a ± 22.9
Length of palisade parenchyma cells (µm)	36.2 b ± 1.32	45.9 a ± 7.42
Number of spongy cell layers	2–3	3
Thickness of spongy layer (µm)	54.3 b ± 8.2	79.7 a ± 1,29
Length of spongy parenchyma cells (µm)	22.7 b ± 3.8	28.6 a ± 4.89
Thickness of adaxial epidermis (µm)	23.8 a ± 4.35	20.4 b ± 2.27
Stomata number in adaxial epidermis	4.0 b ± 0.91	5.0 a ± 1.13
Thickness of abaxial epidermis (µm)	22.1 a ± 4.51	19.1 b ± 2.24
Stomata number in abaxial epidermis	2.1 b ± 0.81	4.6 a ± 1.14

* Values are means ± SD of 10 plants per ecotype from two independent experiments; different letters indicate significantly different means at *p* < 0.05 according to one-way ANOVA and post-hoc Tukey’s test.

**Table 3:** Photosynthetic pigments’ content, phenolic profile as well as radical scavenging activity and lipid peroxidation in leaves of *Anthyllis vulneraria* ecotypes.

	Parameter	Ecotype	
		Metallicolous	Non-Metallicolous
Photosynthetic pigments [mg g^−1^ FW]	Chlorophyll *a*	0.76 a * ± 0.146	0.49 b ± 0.049
	Chlorophyll *b*	0.24 a ± 0.012	0.14 b ± 0.013
	Chlorophyll *a* + *b*	1.01a ± 0.158	0.63 b ± 0.061
	Chlorophyll *a*/*b*	3.10 a ± 0.453	3.46 a ± 0.157
	Carotenoids	0.21 b ± 0.004	0.15 b ± 0.012
Phenolic compounds [mg 100 g^−1^ FW]	Total phenols	1294.21 a ± 20.136	846.69 b ± 36.304
	Phenylpropanoids	365.59 a ± 6.789	249.92 b ± 9.441
	Flavonols	511.35 a ± 14.452	338.94 b ± 13.41
	Anthocyanins	177.61 a ± 14.78	53.42 b ± 9.09
Radical scavenging activity DPPH [%]		20.42 a ± 1.54	20.67 a ± 0.94
Lipid peroxidation level [µmol TBARs g^– 1^ FW]		73.01 a ± 2.73	60.95 b ± 1.12

* Data present means ± SD; different letters indicate significantly different means at *p* < 0.05 according to one-way ANOVA and post-hoc Tukey’s test.

## Abbreviations

AGP	arabinogalactan protein
CAT	catalase
DW	dry weight
EXT	extensin
FW	fresh weight
TMs	trace metals
M	metallicolous ecotype
NM	non-metallicolous ecotype
POX	peroxidase
ROS	reactive oxygen species
SOD	superoxide dismutase
TBARs	thiobarbituric acid-reactive-substances
TEM	transmission electron microscope
TF	translocation factor
